# Effects of ABVD chemotherapy on ovarian function: epidemiology, hormonal dosages and ultrasound morphologic analyses in 270 patients with Hodgkin’s disease

**DOI:** 10.3389/fonc.2023.1059393

**Published:** 2023-04-21

**Authors:** Mariavita Ciccarone, Paola Cavaceppi, Cristiano Tesei, Stefania Brunetti, Alessandro Pulsoni, Ombretta Annibali, Cristiano Gasparoli, Roberta Battistini, Stefan Hohaus, Sabrina Pelliccia, Agostino Tafuri, Maria Christina Cox, Maria Cantonetti, Luigi Rigacci, Elisabetta Abruzzese

**Affiliations:** ^1^Associazione Gemme Dormienti Organizzazione Non Lucrativa di Utilità Sociale (ONLUS), Rome, Italy; ^2^Gynecologic Unit , San Carlo di Nancy Hospital, Rome, Italy; ^3^LabAurelia, Rome, Italy; ^4^Department of Cellular Biotechnology and Haematology, Sapienza University, Rome, Italy; ^5^UOC Haematology Stem Cell Transplantation, University Campus Bio Medico, Rome, Italy; ^6^Laboratory Medicine, San Carlo di Nancy Hospital, Rome, Italy; ^7^UOC Ematologia e Trapianti CSE, Azienda Ospedaliera (AO) San Camillo Forlanini, Rome, Italy; ^8^Policlinico Gemelli Foundation, Catholic University of the Sacred Heart, Rome, Italy; ^9^Haematology Unit, Azienda Ospedaliera‐ Universitaria Sant’Andrea, Rome, Italy; ^10^Hematology, Tor Vergata University Hospital, Rome, Italy; ^11^Hematology, S. Eugenio Hospital, Tor Vergata University, ASL Roma2, Rome, Italy

**Keywords:** fertility preservation, Hodgkin’s lymphoma, ovarian failure, Anti-Mullerian hormone, early menopause

## Abstract

**Introduction:**

Classical Hodgkin Lymphoma (HL) is a lymphoproliferative disease typically diagnosed in the young. The excellent results obtained with current treatment lead to long survival with age-related complications affecting patients’ survival and quality of life. One issue affecting HL patients is infertility. This problem can be easily overcome in males with seminal liquid cryopreservation, however, in females it is more complex either in terms of the quality of the cryopreserved material or the patients’ age at diagnosis. Moreover, not all chemo- or radio-therapies have the same negative impact on fertility.The main objectives of this study was to collect epidemiological information on HL patients involved in fertility preservation counseling and to analyze the impact of ABVD (adriamycin, bleomycin, vinblastine, and dacarbazine), the standard treatment for HL, on ovarian function, hormonal levels and ovarian and uterine tissue morphologies. Patterns of fertility preservation were also reported.

**Methods:**

Data were obtained from 270 female patients at HL onset who were interested in fertility counseling prior to therapy initiation. Each patient was assessed at HL diagnosis for levels of Anti-Mullerian Hormone (AMH), Follicle Stimulating Hormone (FSH), and 17β-oestradiol (17β-oe), with additional assessments at 6 and 12 months after chemotherapy. Patients were evaluated with ultrasound scans to study the number of ovarian follicles and the degree of uterine thickness at the same timepoints.

**Results:**

The average patient AMH level showed a statistically significant reduction at 6 months after chemotherapy (p=0.05) and by the 12 month time point returned to near pre-chemotherapy values. FSH and 17β-oe levels did not significantly vary throughout the study period. ABVD chemotherapy was associated with a significant reduction of both ovarian follicles and endometrial thickness at the 6 month time point followed by a recovery at the 12 time point in both ovaries. Different results were observed when patients changed treatment to a more intensive one.

**Discussion:**

Based on the results from the hormonal measurements and the follicle echography, it appears that the toxic effect of ABVD on fertility is transient, whereas, in contrast, more intensive therapies may potentially be more harmful and long-lasting.

## 1 Introduction

Classical Hodgkin’s Lymphoma (HL) is a common lymphoproliferative disease in young adults. HL has a very good prognosis with chemotherapy and radiotherapy treatments resulting in a high cure rate and long-term survival expectancy. Long-term survival after treatment, however, reveals issues affecting quality of life; one of these is infertility, a major concern in HL patients. In male patients, this problem is avoided with seminal liquid cryopreservation before starting therapy. In female patients, 60% of whom may face infertility due to premature ovarian failure (1), this problem is more complex in terms of the material of cryopreservation or the patient’s age at diagnosis. Many medical articles and studies demonstrate that the type of chemotherapy (alkylating agents in particular) and age at diagnosis are the two pre-eminent risk factors for infertility in reproductive-age woman. Consequently, impacts on reproductive function must be part of the toxicity assessment that both female and male HL patients have to face. Research has confirmed fertility impairment after treatment; women who have undergone therapy are less likely to become pregnant. Information on managing fertility and pregnancy issues is frequently requested by patients and is crucial in their decision-making process (2).

The current “gold standard” treatment in HL is ABVD, theoretically considered to have a low gonadotropic risk (3, 4). For this reason, many young patients diagnosed with HL are not offered oncofertility counseling. Unfortunately, about 15-20% of patients do not respond to first-line therapy (refractory) or relapse and are in need of salvage therapies that are usually more intensive and toxic on the gonads (5). Moreover, some patients develop secondary malignancies, in particular breast cancer (6). No clinical characteristics are observed at diagnosis to identify patients who will not respond or relapse after therapy; FDG-PET has proven useful in identifying negative chemotherapy consequences but only after two ABVD cycles. In clinical practice patients start with ABVD and shift to more intensified therapy with BEACOPP (bleomycin, etoposide, adriamycin, cyclophosphamide, oncovin, procarbazine and prednisone) or autologous stem cell transplantation (ASCT) if interim PET (performed after 2 cycles of ABVD) is positive (7–9). Given these issues, several strategies are currently available for fertility preservation, including ovarian suppression using Gonadotropin-Releasing Hormone agonists (GnRHa) and cryopreservation of oocytes and ovarian tissue (10–14).

The use of GnRHa during chemotherapy (CT) is an attractive option to preserve both ovarian function and fertility, with the advantage of avoiding delays in starting anticancer therapies (6). Compelling evidence supports the benefit yielded by GnRHa administration in cancer patients in terms of reduced risk of CT-induced Premature Ovarian Failure (POF) and increased pregnancy rate with no negative impact on prognosis (15–18). Despite the efficacy of this option in some cancer types, the use of GnRHa remains controversial (19). The Italian Association of Medical Oncology (AIOM) has issued a strong positive recommendation to adopt the use of GnRHa for both ovarian function and FP in cancer patients (20).

Oocyte cryopreservation is now considered a standard option (21, 22). However, it requires controlled ovarian stimulation (COS), that may possibly delay CT start and affect the prognosis of patients with hormone-responsive tumors. To overcome this problem, alternative approaches have been developed, either to avoid COS by cryopreservation of immature oocytes or oocytes matured *in vitro* or to start it at any time using a random start protocol requiring at least two 2 weeks of treatment (23). Ovarian tissue cryopreservation has also been proven to be effective for ovarian function recovery, and since 2019 is no longer been considered experimental (24).

Most importantly, tissue cryopreservation may be performed at any time during the menstrual cycle and is currently the only option available for prepubescent females. Currently, *in vitro* maturation techniques are being investigated to prevent the risk of malignant cell reimplantation associated with tissue cryopreservation (25).

It is highly advisable to suggest fertility preservation options before starting chemotherapy in both women and men if clinical conditions allows it because it is not possible to pre-identify patients who will not respond to standard ABVD and who will require more intensive treatment (8, 9, 26, 27). The ability to recover normal menstrual cycles at the end of treatment does not correlate with fertility. This is very important because there are no current techniques or tests to predict irreversible gonadal damage.

Premature ovarian insufficiency is very low in patients treated with ABVD: in fact, this cycle is considered to have a low impact gonadal toxicity. There are very few reports, however, on the impact of ABVD on patients’ ovarian reserve. Furthermore, there is a paucity of data on hormonal measurements or Antral Follicolar Count (AFC) ultrasound scans to estimate ovarian reserve and other parameters associated with an increased risk of infertility. This retrospective study provides data on ovarian reserve, measuring the levels of three hormones, Anti-Mullerian Hormone (AMH), Follicle Stimulating Hormone (FSH), and 17β-oe at diagnosis and during follow-up. We also studied ovarian function by determining the median number of antral follicles and uterine thickening using ultrasound. These evaluations were simultaneously assessed prior to any preservation techniques.

Several studies suggest AMH may be a useful tool to evaluate female reproductive function (28, 29). AMH regulates folliculogenesis and serves as a reliable biomarker for the relative size of ovarian reserve (30). Monitoring AMH levels provides precise and impartial evaluation of the ovarian reserve at early stages and these data allow for informed decisions and risk adapted strategies which include fertility preservation. AMH information can also be used to assess ovarian depletion after chemotherapy or endocrine therapy in cancer survivors. AMH levels are, therefore, important information for oncologists whose patients are facing pre-menopause due to cancer treatment.

## 2 Patients and methods

Patients in this study were directed to the Gemme Dormienti Association, a member of the Oncofertility Consortium which counsels women with cancer or other diseases that jeopardize fertility about the fertility preservation procedures. Counselling outpatient services, offered by a multi-disciplinary team of medical experts in collaboration with the Association, supplied timely knowledge and awareness to complement treatment and prevent regrets. Patients also received free integrated care including fertility preservation options, ultrasound service and psychological counselling. While fertility preservation is recognized as crucial, providing detailed information and raising awareness on preservation options are not always adopted or deemed important.

Records of patients with a diagnosis of classical HL were obtained from the Gemme Dormienti Association database (26). After a detailed visit and briefing, patients signed an informed consent and were inserted in the database. All patients from January 2013 to June 2020 were included in our analyses. Exclusion criteria for the study were the following: age >40 years old, signs of early ovarian exhaustion, concomitant systemic illnesses, previous chemo- or radiotherapy, the use of birth control pills or other hormonal therapy, or having received pelvic radiotherapy.

Hormonal measurements were performed at HL diagnosis to provide a baseline, and at specific timepoints during follow-up. Patients were monitored according to the type of treatment used: response to therapy, type and length of chemotherapy, and disease evolution. Each woman was evaluated for fertility preservation including assessments of serum AMH, FSH, and 17β-oe levels, at least one ultrasound scan and other tests deemed useful.

Serum AMH levels were determined using an ultrasensitive ELISA. Serum FSH and serum 17β-oe levels were measured by immunoenzymatic method ADVIA Centaur (Siemens Medical Solutions, Tarrytown, NY, USA). All hormonal evaluations were performed at three different time points: 0 (baseline), 6 and 12 months, the latter after the end of chemotherapy. Baseline values were obtained regardless of the phase of menstrual cycle, because therapy initiation is highly advisable as soon as possible after HL diagnosis. The 6 and 12 month hormonal measurements were obtained in consideration of the menstrual cycle, if present. Whenever possible, all assessments – hormonal levels, determination of number of ovarian follicles using ultrasound scans, and uterine thickness – were taken at the same time in the same menstrual cycle, usually in the early postmenstrual phase. Ultrasound scans were performed using a transvaginal probe (Toshiba Aplio 200 until 2015 and then Aplio 500) by a single operator who was not apprised about the results of the hormonal assays. The ultrasound scans assessed the number of antral follicles (all follicles measuring 2-10 mm in mean diameter were counted) and the thickness of the uterus. During the baseline measurements, patients were also evaluated for previous pregnancies or abortions. After treatment they were followed for fertility evaluation, and natural pregnancies, *in vitro* fertilizations and abortions were recorded and entered into the database.

### 2.1 Statistical analysis

Patient data were summarized by means of frequency (n) and percentage (%) for categorical variables and by median (± SD) for continuous variables. Boxplots were used to graphically illustrate AMH, FSH, 17β-oe, AFCr, AFCl and EM values during baseline and follow‐up examinations. Differences among groups were evaluated in univariate analysis by means of non-parametric tests (chi‐squared test for categorical variables, Mann–Whitney and Kruskal–Wallis test for continuous variables, and repeated measures ANOVA for the hormone or echography outcome comparisons which take into account multiple time points).

To standardize menstrual cycles, the duration of each phase within the cycle was fixed. Each phase length was set as follows: menstrual (days 1–5), follicular (days 6–12), ovulatory (days 13–16), luteal (days 17-premenstrual phase). Each variable was expressed as the median per phase. Data obtained in this manner were analyzed through the implementation of statistical methods such as repeated-measures ANOVAs or dependent-sample planned contrasts. If the assumption of sphericity was violated, Greenhouse-Geisser correction was applied. In case of significant interaction or main effects, *post-hoc* tests with Bonferroni correction were performed. All tests were two‐sided, with p <.05 indicating a statistically significant difference. Statistical analyses were performed using RStudio 2021.09.0 + 351 software (31)

## 3 Results

Starting from January 2013 to June 2020, 270 HL patients were referred with the aim of offering fertility preservation counseling and evaluating the impact of induction chemotherapy (ABVD) or intensified salvage therapies on ovarian reserve. The median age was 28.0+/- 6.6 years (range 18.0-40.0); 98 (36.3%) were between 20 and 25 years old, 70 (25.9%) were between 26 and 30 years old, 56 (20.7%) were between 31 and 35 years old and 46 (17.1%) were over 35. Data on 270 patients showed that 186 (68.9%) had no previous pregnancies, 84 (31.1%) had pregnancies with 47 abortions. Two hundred and nineteen (83.5%) had high school or bachelor’s degree. Stage data, based on clinical characteristics, were reported in 160 patients; 10 patients were stage I, 91 stage II, 26 stage III and 33 stage IV. Nearly all patients (266) were treated with the standard ABVD regimen (98.5%); in 4 patients treatment information was unavailable. The vast majority (87%) of those receiving ABVD completed 6 courses. Twenty-five patients (9.5%) were intensified with BEACOPP or salvage treatment with autologous stem cell transplantation due to a positive interim-PET. Eighty-eight patients (33%) were treated with combined chemotherapy and radiotherapy.

Thirty-two patients (11.8%) did not receive fertility preservation, 132 patients (48.9%) were treated with Gonadotrophin Releasing Hormone Agonists (GnRHa) alone, 73 patients (30.7%) were treated with GnRHa after oocytes collection (mean oocyte number was 8.7 +/- 5.5; Range 0-15) and 65 patients (27.3%) were treated with GnRHa after ovarian tissue excision (**Table 1**).

**Table 1:** Demographic and FP characteristics of patients.

Variable n=270		
**Age** (median ys [range] ± SD)		28.0 ± 6.6 (18.0-40.0)
	20-25	36.3% (98/270)
	26-30	25.9% (70/270)
	31-35	20.7% (56/270)
	36+	17.1% (46/270)
**Pregnancies pre therapy** (%)	Yes	68.9% (186/270)
	No	31.1% (84/270
**Abortion pre therapy** (%)	Yes	17.4% (47/270)
	No	82.6% (223/270)
**Ann Arbor Stage**	I	6.3% (10/160)
	II	56.9% (91/160)
	III	16.3% (26/160)
	IV	20.6% (33/160)
**Fertility preservation** (%)	No FP	11.8% (32/270)
	GnRHa	88.2% (238/270)
	GnRHa + oocyte	30.7% (73/238)
	GnRHa + ovarian tissue	27.3% (65/238)

FP, Fertility Preservation; GnRHa, Gonadotrophin-releasing hormone agonists.

Patients who had results on hormonal measurements and ultrasound are reported for eligible patients after screening for which at least 2 timepoint exams were respected.

**Figure 1:**
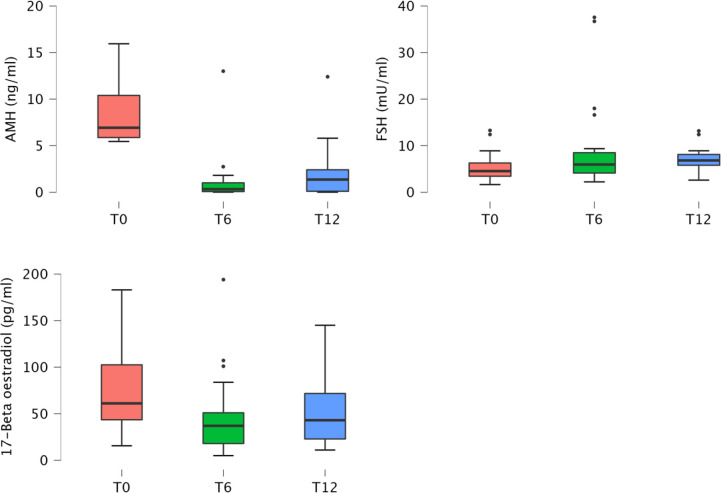
Boxplot of anti‐müllerian hormone, follicle stimulating hormone and 17β-oe levels at baseline and during the follow‐up visits.

AMH, FSH and 17β-oe levels at baseline and during the follow‐up visits are presented in **Table 2** and showed in **Figure 1**.

**Table 2:** AMH, FSH and 17β-oe levels at baseline (overall and according to the menstrual cycle phase) and during the follow‐up visits.

Variable	T0 - median (range)	T6 - median (range)	T12 - median (range)	p-value
**AMH**	n=68	n=62	n=68	
Overall	1.69 (0.0-15.9)	0.56 (0.0-3.9)	1.6 (0.0-5.8)	p < 0.001
Follicular	2.0 0(0.0-10.0)			
Ovulation	2.50 (0.1-9.9)			
Luteal	1.90 (0.0-3.5)			
**FSH**	n=85	n=67	n=85	
Overall	5.8 (0.1-33.4)	7.3 (2.8-31.7)	8.1 (4.6-10-8)	p = 0.019
Follicular	6.1 (0.1-16.0)			
Ovulation	4.6 (0.1-33.4)			
Luteal	4.1 (0.3-14.1)			
**17**β**-oe**	n=82	n=68	n=82	
Overall	64.7 (6.0-384.0)	39.0 (5.0-384.0)	40.0 (8.0-196)	p = 0.005
Follicular	52.5 (6.0-196.0)			
Ovulation	141.0 (41.0-384.0)			
Luteal	93.5 (11.0-384.0)			

AMH, anti‐müllerian hormone; FSH, follicle‐stimulating hormone; 17β-oe, 17 Beta-oestradiol.

Considering all patients, mean AMH levels showed a reduction at the 6 month time point, then increased with a statistically significant difference (p < 0.001) during the follow-up, almost returning to pre-chemotherapy values (12 months; **Table 2**; **Figure 1**). FSH levels increased (p=0.019) and 17β-oe levels decreased (p=0.005) at both post-baseline time points and its values preserve normal levels without significant differences during follow-up (**Table 2**). Comparing patients treated with ABVD and patients intensified, we observed a difference in the expression of FSH that increased in patients treated with intensified treatment (p not significant) and a trend for AMH value (p not significant) that did not improve during follow-up in patients intensified. This is not statistically significant probably due to the low number of evaluable samples, but is strongly suggesting the severity of damage in such subset (**Table 2**).

Morphological analysis of ovarian follicles and uterine thickness, evaluated with ultrasonography, showed very interesting results in the whole group of patients. ABVD chemotherapy was associated with a significant reduction of follicles at 6 months and a recovery at the 12 month time point in both ovaries (**Table 3**). Interestingly, endometrium thickness decreased at 6 months and recovered to basal values at 12 months (**Table 3**). Considering the treatment used, we can observe, even if in a low number of patients, that in the intensified group during follow-up the number of follicles was 0 in both ovaries and the endometrium thickness is very low (**Table 4**). Obviously these data with a longer follow-up could change.

**Table 3:** Ecography outcome at baseline and during the follow‐up visits.

Ecography variables	T0 - median ± SD (range)	T6 - median ± SD (range)	T12 - median ± SD (range)	p-value
**AFCr**	n=42	n=31	n=42	p < 0.001
	7.0 ± 3.4 (0.0-16.0)	1.0 ± 1.7 (0.0-4.0)	3.0 ± 2.5 (0.0-8.0)	
**AFCl**	n=41	n=31	n=41	p < 0.001
	6.0 ± 5.2 (0.0-33.0)	1.0 ± 2.1 (0.0-5.0)	7.0 ± 2.2 (3.0-9.0)	
**EM (mm)**	n=48	n=32	n=48	p = 0.002
	7.5 ± 3.6 (0.0-14.0)	2.0 ± 1.7 (1.0-5.4)	4.7 ± 2.1 (1.2-7.6)	

AFCr, antral follicular count right; AFCl, antral follicular count left; EM Endometrial thickness.

**Table 4:** Hormonal and ecography variables in the ABVD vs intensification chemotherapy patients at baseline and during the follow‐up visits.

Hormone variables	T0 - median (range)	T6 - median (range)	T12 - median (range)	p-value	
AMH					
ABVD	n= 58	n=58	n=51	p = ns	
	6.7 (5.4-15.9)	0.6 (0.0-13.0)	2.2 (0.0-12.4)		
Intensification	n= 9	n=6	n=9		
	7.2 (5.5-10.6)	0.1 (0.0-1.0)	0.0 (0.0-3.2)		
FSH					
ABVD	n= 76	n=41	n=76	p = ns	
	4.3(1.6-12.4)	5.9 (2.2-8.5)	7.0 (2.6-13.2)		
Intensification	n= 9	n=6	n=9		
	8.9 (5.3-13.3)	37.1 (16.6-61.8)	74.8 (4.9-90.2)		
17β-oe					
ABVD	n= 73	n=63	n=73	p = ns	
	60.1 (15.6-183.0)	39.0 (11.8-385.0)	48.0 (11.0-128.0)		
Intensification	n= 9	n=5	n=9		
	64.7 (52.4-112.0)	30.0 (5.0-107.0)	25.0 (11.8-145)		
Ecography variables					
AFCr					
ABVD	n=36	n=28	n=36		p = ns
	8.0 ± 3.3 (0.0-12.0)	0.0 ± 2.3 (0.0-6.0)	3.0 ± 4.4 (0.0-19.0)		
Intensification	n= 8	n=3	n=8		
	7.0 ± 4.1 (2.0-16.0)	0.0 ± 1.5 (0.0-3.0)	2.0 ± 2.4 (0.0-7.0)		
AFCl					
ABVD	n=36	n=28	n=36		p = ns
	6.0 ± 3.2 (2.0-15.0)	1.0 ± 2.3 (0.0-5.0)	4.0 ± 7.1 (0.0-33.0)		
Intensification	n= 8	n=3	n=8		
	5.5 ± 2.2 (3.0-9.0)	2.0 ± 2.6 (0.0-5.0)	4.0 ± 3.4 (0.0-8.0)		
EM (mm)					
ABVD	n=36	n=28	n=36		p = ns
	7.5 ± 3.9 (1.7-14.0)	2.9 ± 4.1 (0.0-13.0)	5.0 ± 2.8 (1.0-12.0)		
Intensification	n= 8	n=3	n=8		
	7.2 ± 2.6 (3.0-11.0)	2.5 ± 1.8 (1.0-5.4)	3.2 ± 1.5 (2.0-14.0)		

AFCr, antral follicular count right; AFCl, antral follicular count left; EM Endometrial thickness.

We observed 9, post-chemotherapy pregnancies in our study group, all spontaneous with seven natural deliveries and one cesarean. All were healthy babies. One pregnancy ended in an elective abortion due to the temporal closeness of chemotherapy and radiotherapy. Information on how many patients tried to get pregnant after therapy was not available. However, if conception was difficult, patients usually were referred again for eventual treatment.

## 4 Discussion

The ability to conceive after treatment represents a major concern for young, cured lymphoma patients. The high incidence of HL diagnosis in young people and a much longer life in these patients due to the high success rate of therapy have resulted in the emergence of quality of life issues during and after treatment.

The complex equilibrium between life-saving treatment and short/long-term toxicity is not easily balanced. For many young patients, fertility is one of the most important issues to be considered when deciding on a treatment. Unfortunately, there are few published reports on the impact of different treatments on gonadal function to guide clinicians and their patients. For example, although fertility preservation is easily accomplished in males using seminal liquid cryopreservation before starting chemotherapy, ensuring ongoing fertility in female patients is much more complicated and less well known. Fertility issues are dramatically different in female patients because cryopreservation of oocytes or ovarian tissue is not as easy. The issue is further complicated by the toxic effects of chemotherapy on gonadal function in women.

Chemotherapy-induced toxic effects on ovaries are well known although it is still not clear if they are always associated with infertility. Several lines of evidence indicate that gonadal function in females after ABVD may not be severely impacted. ABVD therapy is associated with a very low incidence of chemotherapy-induced amenorrhea, infertility or premature ovarian failure (32). For this reason, recent recommendations for fertility preservation in HL patients have suggested that women < 30 yrs treated with ABVD should be informed of their good fertility prognosis and could be treated with GnRHa even if there isn’t a unanimous consensus about protection on ovarian function (33, 34). One caveat is that 20-25% of patients treated with ABVD at diagnosis do not respond to therapy or relapse early and need salvage therapy with intensified treatments having gonadotoxic side effects (35). Those gonadotoxic intensified therapies present a high risk of inducing early menopause requiring infertility treatments. Thus, fertility issues and preservation methods should be discussed with women even if a low gonadotoxic treatment is considered (36).

Cryopreservation of ovarian tissue or oocytes should be attempted prior to chemotherapy as these preservation techniques are not yet recommended after chemotherapy exposure. However, it must be noted that there are few situations, including high tumor burden, mass at pelvic site, lack of preservation at diagnosis and need for intensification, in which fertility preservation before treatment cannot be done. A recent report on 25 individuals, including 16 HL patients, treated with chemotherapy before ovarian tissue cryopreservation revealed that ovarian function recovery and pregnancy incidence compared favorably with those reported in patients not treated before preservation (37). Thus selected cases could be identified and discussed multidisciplinarly.

A secondary analysis of the RATHL study showed that the recovery of ovarian function after ABVD treatment was dependent on age (38). This is supported by the observation that a full recovery of AMH levels was observed in younger patients less than 35 years but not in older ones, even though AMH levels declined in all patients, young and older, gathering that ABVD chemotherapy was almost harmless in patients under 35 (39). This conclusion is interesting in light of the study by Hodgson et al. (40), which showed that ovarian reserve is reduced in female patients with HL diagnosis even before starting chemotherapy. Azem et al. (41), verified that the risk of ovarian failure or damage was very low in HL patients, however, in this study the pre-CT AMH level was not provided.

In our study 270 patients with diagnosis of HL were registered in the Gemme Dormienti database and several parameters including gonad-regulating hormone levels, uterine thickness and follicle number were measured before and after treatment to evaluate fertility balance and to guide the choices on fertility preservation. In this pivotal study we found no differences in hormonal recovery in different age groups. Unfortunately, not all patients could be fully studied during follow-up at the end of treatment. Although limited, our data showed interesting results particularly for the AMH trend in before and after treatment and for the anatomical modification of ovaries and uterus during therapy.

AMH levels are sensitive to ovarotoxic effects of chemotherapy agents and remains the best substitute marker of gonadal toxicity even if caution is still required. Timing of AMH measurements needs to be further refined due to differences in treatment regimens. In our study AMH measurements 6 and 12 months after chemotherapy completion proved informative of recovery after a standard ABVD regimen (42). It has been shown that post-chemotherapy AMH levels are significantly lower, due to AMH-producing early-stage follicles damaged by all forms of chemotherapy (43, 44).

New growth by surviving follicle reserves with *de novo* AMH production indicates partial recovery of AMH levels, and represents a new state of the ovarian reserve. In addition, ultrasound analysis of antral follicle count can also be considered a possible surrogate marker to monitor the reserve of each ovary separately, providing a non-invasive and easy protocol to screen and follow patients. Thus, the hormonal measurements and echography results presented in this paper suggest that the toxic effects of ABVD on fertility is transient, in contrast to the gonadotoxic effects of treatment intensification which might be more substantial and long-lasting.

To our knowledge this is the largest and most detailed study on the effects of ABVD and more intensive regimens on ovarian function in young HL patients. Our results confirm the data observed in the RATHL study on AMH levels in ABVD-treated patients. Very interestingly, in our opinion, is the observation of disappearance of follicles one year after the end of chemotherapy in all patients treated with intensification therapy. The limited number of patients in this group (8-9/25) precludes a definitive conclusion and further research on this issue is in progress.

The data presented here underline the need to discuss on fertility preservation with all patients regardless of the risk of gonadal failure. In women, different options for fertility preservation exist, and the specific approach chosen depends on multiple factors including the patient’s age, the parity, urgency to start therapy, personal situation, marital status and patient’s wishes. Securing fertility before chemotherapy should have a high priority given its psychological and social impact (45, 46).

The lower number of patients completing the study, compared to the enrolled population group, points to the need of reinforcing the compliance both in patients successfully completing their treatment, and in the group needing intensification.

## 5 Conclusions

Patients of reproductive age diagnosed with cancer often face fertility impairment. Increased awareness of the factors underlying fertility preservation is of the utmost importance to support and guide them in their decision-making process. It is every woman’s right to be informed about the availability of fertility preservation procedures. Providing this information to as many women and physicians as possible, in order to avoid decisional regret and its consequences, is a major aim of the Gemme Dormienti Association through its extensive counseling and network connecting oncological-hematological and reproductive preservation centers throughout Italy. Without detailed fertility preservation information, the patient’s quality of life may be negatively affected. For this reason, the informed decision-making process must be considered a pivotal part of the treatment. Unfortunately, this type of approach, while recognized as important, is still not being implemented with potentially negative consequences to uninformed patients.

## Data availability statement

The raw data supporting the conclusions of this article will be made available by the authors, without undue reservation.

## Ethics statement

Ethical review and approval was not required for the study on human participants in accordance with the local legislation and institutional requirements. The patients/participants provided their written informed consent to participate in this study.

## Author contributions

MCi conceived the study and made significant contributions to the study methods, results, and interpretation. LR and EA were involved in the design, writing and critically revised the content of this manuscript and share last authorship. All the authors contributed to data harvest, to manuscript conception and writing. All authors contributed to the article and approved the submitted version.

## References

[1] Kiserud CE Fossa A Holte H Fossa SD. Post-treatment parenthood in hodgkin’s lymphoma survivors. Br J Cancer (2007) 96:1442–14493. doi: 10.1038/sj.bjc.6603711 17406362 PMC2360165

[2] Hutchcraft ML McCracken K Whiteside S Lustberg M Lindheim SR Nahata L. Current fertility preservation options for female patients with Hodgkin lymphoma. Obstet Gynecol Surv (2020) 75(11):683–91. doi: 10.1097/OGX.0000000000000835 33252698

[3] Schmidt KT Andersen CY ISFP Practice Committee. Recommendations for fertility preservation in patients with lymphomas. J Assist Reprod Genet (2012) 29(6):473–7. doi: 10.1007/s10815-012-9787-x PMC3370035 22562284

[4] Brusamolino E Lunghi F Orlandi E Astori C Passamonti F Baraté C. Treatment of early-stage hodgkin’s disease with four cycles of ABVD followed by adjuvant radio therapy: analysis of efficacy and long-term toxicity. Haematologica (2000) 85(10):1032–9. 11025593

[5] Behringer K Thielen I Mueller H Goergen H Eibl AD Rosenbrock J. Fertility and gonadal function in female survivors after treatment of early unfavorable Hodgkin lymphoma (HL) within the German Hodgkin study group HD14 trial. Ann Oncol (2012) 23(7):1818–25. doi: 10.1093/annonc/mdr575 22228451

[6] Cooke R Jones ME Cunningham D Falk SJ Gilson D Hancock BW. Breast cancer risk following Hodgkin lymphoma radiotherapy in relation to menstrual and reproductive factors. Br J Cancer. (2013) 108(11):2399–2406. doi: 10.1038/bjc.2013.219 23652303 PMC3681009

[7] Rigacci L Castagnoli A Carpaneto A Carrai V Vaggelli L Matteini M. Can (18)F-FDG PET after first cycle chemotherapy predict the efficacy of therapy in hodgkin's disease? Haematologica (2002) 87(5):ELT24. doi: 10.3324/%x 12010684

[8] Gallamini A Rigacci L Merli F Nassi L Bosi A Capodanno I. The predictive value of positron emission tomography scanning performed after two courses of standard therapy on treatment outcome in advanced stage hodgkin's disease. Haematologica (2006) 91(4):475–81. 16585014

[9] Gallamini A Hutchings M Rigacci L Specht L Merli F Hansen M. Early interim 2-[18F]fluoro-2-deoxy-D-glucose positron emission tomography is prognostically superior to international prognostic score in advanced-stage hodgkin's lymphoma: a report from a joint Italian-Danish study. J Clin Oncol (2007) 25(24):3746–52. doi: 10.1200/JCO.2007.11.6525 17646666

[10] Blondeaux E Massarotti C Fontana V Poggio F Arecco L Fregatti P. The PREgnancy and FERtility (PREFER) study investigating the need for ovarian function and/or fertility preservation strategies in premenopausal women with early breast cancer. Front Oncol (2021) 11:690320. doi: 10.3389/fonc.2021.690320 34150661 PMC8210666

[11] Domingo J Garcia-Velasco JA. Oocyte cryopreservation for fertility preservation in women with cancer. Curr Opin Endocrinol Diabetes Obes (2016) 23:465–9. doi: 10.1097/MED.0000000000000295 27685935

[12] Lambertini M Del Mastro L Pescio MC Andersen CY Azim HA Jr Peccatori F. Cancer and fertility preservation: International recommendations from an expert meeting. BMC Med (2016) 14. doi: 10.1186/s12916-015-0545-7 PMC4700580 26728489

[13] Deshpande NA Braun IM Meyer FL. Impact of fertility preservation counseling and treatment on psychological outcomes among women with cancer: A systematic review. Cancer (2015) 15:3938–47. doi: 10.1002/cncr.29637 26264701

[14] De Vos M Smitz J Woodruff TK. Fertility preservation 2: fertility preservation in women with cancer. Lancet (2014) 384(9950):1302–10. doi: 10.1016/S0140-6736(14)60834-5 PMC4270060 25283571

[15] Blumenfeld Z Evron A. Endocrine prevention of chemotherapy-induced ovarian failure. Curr Opin Obstetrics Gynecol (2016) 28(4):223–9. doi: 10.1097/GCO.0000000000000278 27253235

[16] Del Mastro L Boni L Michelotti A Gamucci T Olmeo N Gori S. Effect of the gonadotropin-releasing hormone analogue triptorelin on the occurrence of chemotheraopy-induced early menopause in premenopausal women with breast cancer: a randomized trial. JAMA (2011) 306(3):269–76. doi: 10.1001/jama.2011.991 21771987

[17] Del Mastro L Ceppi M Poggio F Bighin C Peccatori F Demeestere I. Gonadotropin-releasing hormone analogues for the prevention of chemotherapy-induced premature ovarian failure in cancer women: systemic review and meta-analysis of randomized trials. Cancer Treat Rev (2014) 40(5):675–83. doi: 10.1016/j.ctrv.2013.12.001 24360817

[18] Lambertini M Horicks F Del Mastro L Partridge AH Demeestere I. Ovarian protection with gonadotropin-releasing hormone agonists during chemotherapy in cancer patients: from biological evidence to clinical application. Cancer Treat Rev (2019) 72:65–77. doi: 10.1016/j.ctrv.2018.11.006 30530271

[19] Demeestere I Brice P Peccatori F Kentos A Dupuis J Zachee P. No evidence for the benefit of gonadotropin-releasing hormone agonist in preserving ovarian function and fertility in lymphoma survivors treated with chemotherapy: final long-term report of a prospective randomized trial. J Clin Oncol (2016) 34(22):2568–74. doi: 10.1200/jco.2015.65.8864 27217453

[20] Lambertini M Cinquini M Moschetti I Peccatori F Anserini P Valenzano Menada M. Temporary ovarian suppression during chemotherapy to preserve ovarian function and fertility in breast cancer patients: a GRADE approach for evidence evaluation and recommendations by the Italian association of medical oncology. Eur J Cancer. (2017) 71:25–33. doi: 10.1016/j.ejca.2016.10.034 27940355

[21] Viviani S Caccavari V Gerardi C Ramadan S Allocati E Minoia C. Male And female fertility: Prevention and monitoring hodgkin' lymphoma and diffuse Large b-cell lymphoma adult survivors. a systematic review by the fondazione italiana linfomi. Cancers (Basel) (2021) 13(12):2881. doi: 10.3390/cancers13122881 34207634 PMC8228520

[22] Øvlisen AK Jakobsen LH Eloranta S Kragholm KH Hutchings M Frederiksen H. Parenthood rates and use of assisted reproductive techniques in younger Hodgkin lymphoma survivors: A Danish population-based study. J Clin Oncol (2021) 39(31):3463–72. doi: 10.1200/JCO.21.00357 34170749

[23] ISFP Practice Committee Kim SS Donnez J Barri P Pellicer A Patrizio P. Recommendations for fertility preservation in patients with lymphoma, leukemia and breast cancer. J Assisted Reprod Genet (2012) 29(6):465–8. doi: 10.1007/s10815-012-9786-y PMC3370045 22648282

[24] Practice Committee of the American Society for Reproductive Medicine. Fertility preservation in patients undergoing gonadotoxic therapy or gonadectomy: a committee opinion. Fertil Steril. (2019) 112(6):1022–33. doi: 10.1016/j.fertnstert.2019.09.013 31843073

[25] Virant-Klun I Bauer C Stahlberg A Kubista M Skutella T. Human oocyte maturation *in vitro* is improved by co-culture with cumulus cells from mature oocytes. Reprod BioMed Online. (2018) 36(5):508–23. doi: 10.1016/j.rbmo.2018.01.011 29503212

[26] Ciccarone M Hohaus S Pulsoni A Cavaceppi P Franzò S Fabbri R. Preliminary results of a counselling programme for fertility preservation in female cancer patients: The experience of the GEMME DORMIENTI network. Eur J Cancer Care (Engl) (2020) 29(1):e13174. doi: 10.1111/ecc.13174 31571303

[27] Amzai G Karanfilski O Stavrikj SG Stojanovikj A. Reproductive issues in long-term surviving patients following therapy for hodgkin's disease in the republic of north Macedonia: Risks of infertility according to first-line treatment regimens. Hematol Rep (2022) 14(2):85–94. doi: 10.3390/hematolrep14020013 35466177 PMC9036275

[28] Dunlop CE Anderson RA. Uses of anti-mullerian hormone (AMH) measurement before and after cancer treatment in women. Maturitas (2015) 80:245–50. doi: 10.1016/j.maturitas.2014.12.005 25596814

[29] Anderson RA Rosendahl M Kelsey TW Cameron DA. Pretreatment anti-mullerian hormone predicts for loss of ovarian function after chemotherapy for early breast cancer. Eur J Cancer. (2013) 49(16):3404–11. doi: 10.1016/j.ejca.2013.07.014 PMC3807650 23968732

[30] Decanter C Morschhauser F Pigny P Lefebvre C Gallo C Dewailly D. Anti-mullerian hormone follow-up in young women treated by chemotherapy for lymphoma: preliminary results. Reprod BioMed Online. (2010) 20(2):280–5. doi: 10.1016/j.rbmo.2009.11.010 20113967

[31] RStudio team. RStudio: integrated development for RStudio, PBC, Boston, MA (2020). Available at: http://www.rstudio.com/.

[32] Falorio S Biasoli I Luminari S Quintana G Musso M Dell'olio M. Risk factors for impaired gonadal function in female Hodgkin-lymphoma survivors: final analysis of a retrospective multicenter joint study from Italian and Brazilian institutions. Hematol Oncol (2013) 31(2):72–8. doi: 10.1002/hon.2029 23027689

[33] Behringer K Wildt L Mueller H Mattle V Ganitis P van den Hoonaard B. No protection of the ovarian follicle pool with the use of GnRH-analogues or oral contraceptives in young women treated with escalated BEACOPP for advanced-stage Hodgkin lymphoma. final results of a phase II trial from the German Hodgkin study group. Ann Oncol (2010) 21(10):2052–60. doi: 10.1093/annonc/mdq066 20305034

[34] Dolmans MM Taylor HS Rodriguez-Wallberg KA Blumenfeld Z Lambertini M von Wolff M. Utility of gonadotropin-releasing hormone agonists for fertility preservation in women receiving chemotherapy: pros and cons. Fertil Steril. (2020) 114(4):725–38. doi: 10.1016/j.fertnstert.2020.08.011 33040981

[35] Behringer K Mueller H Goergen H Thiele I Eibl AD Stumpf V. Gonadal function and fertility in survivors after Hodgkin lymphoma treatment within the German Hodgkin study group HD13 to HD15 trials. J Clin Oncol (2013) 31(2):231–9. doi: 10.1200/JCO.2012.44.3721 23150709

[36] Jadoul P Kim SS ISFP Practice Committee. Fertility considerations in young women with hematological malignancies. J Assist Reprod Genet (2012) 29(6):479–87. doi: 10.1007/s10815-012-9792-0 PMC3370036 22614159

[37] Poirot C Fortin A Dhédin N Brice P Socié G Lacorte JM. Post-transplant outcome of ovarian tissue cryopreserved after chemotherapy in hematologic malignancies. Haematologica (2019) 104(8):e360–3. doi: 10.3324/haematol.2018.211094 PMC6669155 30765476

[38] Anderson RA Remedios R Kirkwood AA Patrik P Steves L Clifton-Hadley L. Determinants of ovarian function after response-adapted therapy in patients with advanced hodgkin’s lymphoma (RATHL): a secondary analysis of a randomised phase 3 trial. Lancet Oncol (2018) 19(10):1328–37. doi: 10.1016/S1470-2045(18)30500-X PMC6167406 30220622

[39] Policiano C Subirà J Aguilar A Monzò S Iniesta I Rubio JM. Impact of ABVD chemotherapy on ovarian reserve after fertility preservation in reproductive-aged women with Hodgkin lymphoma. J Assisted Reprod Genet (2020) 37(7):1755–61. doi: 10.1007/s10815-020-01844-0 PMC7376990 32488563

[40] Hodgson DC Pintilie M Gitterman L Dewitt B Buckley CA Ahmed S. Fertility among female Hodgkin lymphoma survivors attempting pregnancy following ABVD chemotherapy. Hematol Oncol (2007) 25(1):11–5. doi: 10.1002/hon.802 17036376

[41] Azem F Samara N Cohem T Bem-Yosef D Almog B Lessing BJ. Assessment of ovarian reserve following ovarian tissue banking and/or GnRH-a co-treatment prior to chemotherapy in patients with hodgkin’s disease. J Assist Reprod Genet (2008) 25(11-12):535–8. doi: 10.1007/s10815-008-9276-4 PMC2593773 19015974

[42] Decanter C Delepine J Behal H Manier S Bruno B Barbatti M. Longitudinal study of AMH variations in 122 adolescents and young adults (AYA) and non-AYA lymphoma patients to evaluate the chemo-induced ovarian toxicity to further personalise fertility preservation counselling. Hum Reprod (2021) 36(10):2743–52. doi: 10.1093/humrep/deab189 34417822

[43] Kongkiatkamon S Chintabanyat A Polprasert C Uaprasert N Rojnuckarin P. Post-treatment anti-mullerian hormone (AMH) levels predict long-term ovarian dysfunction in women with hematological malignancies. Hematology (2022) 27(1):181–6. doi: 10.1080/16078454.2022.2026018 35068389

[44] Donnez J Dolmans MM. Fertility preservation in women. N Engl J Med (2017) 377(17):1657–65. doi: 10.1056/NEJMra1614676 29069558

[45] Cacciottola L Donnez J Dolmans MM. Ovarian tissue and oocyte cryopreservation prior to iatrogenic premature ovarian insufficiency. Best Pract Res Clin Obstet Gynaecol. (2022) 81:119–33. doi: 10.1016/j.bpobgyn.2021.09.010 34887172

[46] Broer SL Broekmans FJM Laven JSE Fauser BCJM. Anti-mullerian hormone: ovarian reserve testing and its potential clinical implications. Hum Reprod Update. (2014) 20(5):688–701. doi: 10.1093/humupd/dmu020 24821925

